# Diarrheal bacterial pathogens and multi-resistant enterobacteria in the Choqueyapu River in La Paz, Bolivia

**DOI:** 10.1371/journal.pone.0210735

**Published:** 2019-01-14

**Authors:** Jessica Guzman-Otazo, Lucia Gonzales-Siles, Violeta Poma, Johan Bengtsson-Palme, Kaisa Thorell, Carl-Fredrik Flach, Volga Iñiguez, Åsa Sjöling

**Affiliations:** 1 Instituto de Biología Molecular y Biotecnología, Universidad Mayor de San Andrés, La Paz, Bolivia; 2 Department of Microbiology, Tumor and Cell Biology, Karolinska Institutet, Stockholm, Sweden; 3 Department of Infectious Diseases, Institute of Biomedicine, Sahlgrenska Academy, University of Gothenburg, Gothenburg, Sweden; 4 Centre for Antibiotic Resistance Research (CARe) at the University of Gothenburg, Gothenburg, Sweden; 5 Wisconsin Institute for Discovery, University of Wisconsin-Madison, Madison, WI, United States of America; Georgetown University, UNITED STATES

## Abstract

Water borne diarrheal pathogens might accumulate in river water and cause contamination of drinking and irrigation water. The La Paz River basin, including the Choqueyapu River, flows through La Paz city in Bolivia where it is receiving sewage, and residues from inhabitants, hospitals, and industry. Using quantitative real-time PCR (qPCR), we determined the quantity and occurrence of diarrheagenic *Escherichia coli* (DEC), *Salmonella enterica*, *Klebsiella pneumoniae*, *Shigella spp*. and total enterobacteria in river water, downstream agricultural soil, and irrigated crops, during one year of sampling. The most abundant and frequently detected genes were *gapA* and *eltB*, indicating presence of enterobacteria and enterotoxigenic *E*. *coli* (ETEC) carrying the heat labile toxin, respectively. Pathogen levels in the samples were significantly positively associated with high water conductivity and low water temperature. In addition, a set of bacterial isolates from water, soil and crops were analyzed by PCR for presence of the genes *bla*_CTX-M_, *bla*_KPC,_ *bla*_NDM_, *bla*_*VIM*_ and *bla*_*OXA-48*._ Four isolates were found to be positive for *bla*_CTX-M_ genes and whole genome sequencing identified them as *E*. *coli* and one *Enterobacter cloacae*. The *E*. *coli* isolates belonged to the emerging, globally disseminated, multi-resistant *E*. *coli* lineages ST648, ST410 and ST162. The results indicate not only a high potential risk of transmission of diarrheal diseases by the consumption of contaminated water and vegetables but also the possibility of antibiotic resistance transfer from the environment to the community.

## Introduction

Diarrheal diseases are a major cause of morbidity and mortality worldwide with particular impact on children [1, 2]. In Bolivia, recent estimates suggest that 21% of children under 5 years of age suffer from acute diarrhea at least once a year [3], and that acute diarrhea is responsible for approximately 15% of the annual total number of deaths in Bolivian children less than 5 years old, entailing a very high cost for health systems and involved families [4].

The main diarrheal bacterial pathogens are often water- and/or food-borne and infect through ingestion of contaminated sources. Diarrheal pathogens have been shown to persist over long periods of time in natural water reservoirs and sediments [5, 6]. Moreover, some diarrheal pathogens are also able to attach to fresh produce, especially lettuce and other vegetables [7]. Hence, contaminated drinking and irrigation water may be one of the major sources of infection. The La Paz river basin, with the major river called Choqueyapu, flows from the mountains above the Altiplano at 4100 MASL and crosses La Paz city. The Choqueyapu River frequently receives household, industrial and hospital drainage without any previous treatment [8–10]. Therefore, the population of La Paz is directly and indirectly exposed to the contaminated Choqueyapu River. In addition, further down the course of the river in the agricultural region of Mecapaca, river water is used for irrigation and river sediments as fertilizers for crops that supply vegetables to the population of La Paz and the nearby city El Alto [10]. In La Paz, consumption of fresh produce bought at local markets and cultured in the more tropical parts of the city has been associated with outbreaks of diarrhea [9].

In urban settings, water bodies and soils may receive heavy discharges of contaminants, including fecal bacteria from human and non-human origin, which also may carry antibiotic resistance genes. In that sense, water bodies and aquatic sediments can support the dissemination of antibiotic resistant bacteria [11]. In addition, discharges of antibiotics and other antimicrobials from anthropogenic sources might select for and further promote dissemination of antibiotic resistance genes and resistant bacteria in these environments [12].

The aim of this study was to analyze the occurrence and bacterial load of diarrheal pathogens in water, soil and vegetable samples from the Choqueyapu area and affluent rivers in the La Paz River basin by molecular methods. In addition, a subset of bacterial strains isolated from river water and the agricultural area, and found to be resistant for at least 3 antibiotics by disc diffusion tests, were analysed by PCR for presence of genes encoding selected extended spectrum β lactamases (ESBL) and carbapenemases, which are important antibiotics in treatment of infections.

## Materials and methods

### Study area and sampling points

The Choqueyapu River which is part of the La Paz River basin originates in the Pampalarama lagoon, a mountainous area in the north part of the city. The Choqueyapu River crosses the La Paz city receiving discharge of wastewater from urban and industrial sources. This urban river continues to the south part of the city where it merges with the tributary rivers Irpavi and Lakha Kollu to form the La Paz River. In this study, we used molecular techniques to evaluate the same set of samples as in the previously published work by Poma et al. [10], where water, soil and vegetable samples were collected and cultured once per month, during one year (2013–14) at different points of the La Paz River basin. Samples from January 2014 were not included in the present study. The sampling points were the first point (SP1)- Incachaca- located at the exit point of a fresh water reservoir (16°24'26.19"S, 68°02'54.68"W; 4147 MASL); the second point (SP2)- Holguín- located in La Paz city, close to hospital and municipal wastewater discharges (16°31'19.99"S, 68°06'55.47"W; 3183 MASL), the third point (SP3)- Mecapaca- at the agricultural region where all wastewater generated at the city is collected and used for irrigation of crops (16°40'11.77"S, 68°01'38.19"W; 2865 MASL) and the last point (SP4)- Jillusaya River- an affluent of La Paz River impacted by municipal wastewater discharges (16°32'12.54"S, 68°04'00.89"W; 3226 MASL). The detailed map of the four sampling points can be found elsewhere [10].

### Sample collection

The sample collection was performed during the study by Poma et al. [10]. Briefly, 200 mL of river water was collected from each sampling point and filtered onto 0.45 μm porosity filters (Millipore). Due to occasional clogging of the filters in contaminated samples, the exact filtered volumes were recorded for each sample and subsequently used for calculations of absolute numbers of bacteria per volume (100 mL). In the case of vegetables, 30 g of collected lettuce or chard were rinsed in 200 mL of peptone water, which then was filtered onto 0.45 μm porosity filters (Millipore). Filters were then cut with sterile razor blades into halves or quarters and stored at -70°C prior to DNA extraction. In the case of soil, 200 mg were collected in a sterile 1.5 mL tube and stored at -70°C until DNA extraction. Vegetables and soil were only taken in SP3, the agricultural area.

No specific permissions were required for water samples from the Choqueyapu River and the La Paz River basin sampling points because they are not part of any national park, protected area or private land. An oral permission from farmers was acquired for vegetable and soil samples. Field studies did not involve endangered or protected species

### DNA extraction

A quarter of a filter (river water or vegetables rinse water) and 200 mg of soil samples were added into tubes containing 1 mL of Inhibitex Buffer (Qiagen GmbH, Helden, Germany). The contents were sonicated on ice for one to two minutes in order to loosen attached bacteria from filters and soil particles. DNA was then extracted using Qiagen’s Fast DNA Stool Mini Kit (Qiagen GmbH, Helden, Germany) according to the manufacturer’s protocol. The DNA was eluted in 200 μL of Elution Buffer and stored at -20°C. The concentration was determined by measuring the OD_260_ in a NanoDrop spectrophotometer (NanoDrop Technologies, Wilmington, DE, USA).

### Detection of diarrheal bacterial pathogens

qPCR Primers were selected from previous publications, the pathogen detected, target gene, primer sequences, product size, and references are listed in Table 1. The specificity of all primers was tested by BLAST analysis (https://blast.ncbi.nlm.nih.gov) using both primers. Correct amplification was determined using both positive and negative reference strains for each respective pathogen. The size and quality of all PCR products were verified by using reference strain DNA and standard PCR conditions. The products were separated on agarose gels and visualised by GelRed (Biotium Inc., California, USA) under UV light. PCR products used for standard curves were purified using the PCR Purification Kit (Qiagen GmbH, Helden, Germany).

**Table 1:** List of oligonucleotide primers for real-time PCR detection of diarrheal bacterial pathogens.

Pathogen	Target Gene	Primers sequence (5’→3’)	Product Size (bp)	Reference
*Enterobacteriaceae*	Glyceraldehyde 3-phosphate dehydrogenase-A (*gapA*)	F: CGTTGAAGTGAAAGACGGTCATC R: CAACACCAACTTCGTCCCATTT	101	[13]
*Enterotoxigenic Escherichia coli (ETEC)*	Heat-labile toxin B chain (e*ltB*)	F: GGCAGGCAAAAGAGAAATGG R: TCCTTCATCCTTTCAATGGCT	117	[14]
*Enterotoxigenic Escherichia coli (ETEC)*	Heat-stable toxin (*estA1*)	F: TCTTTCCCCTCTTTTAGTCAG R: ACAGGCAGGATTACAACAAAG	166	[15]
*Enterotoxigenic Escherichia coli (ETEC)*	Heat-stable toxin (*estA2-4)*	F: TTCACCTTTCCCTCAGGATG R: CTATTCATGCTTTCAGGACCA	120	[16]
*Enteropathogenic Escherichia coli (EPEC)/* *Enterohemorrhagic Escherichia coli (EHEC)*	Intimin (*eae*)	F: GCTATAACRTCTTCATTGATC R: RCTACTTTTRAAATAGTCTCG	92	[17]
*Enteroaggregative Escherichia coli (EAEC)*	Enteroaggregative regulator (*aggR*)	F: TTTATCGCAATCAGATTAARC R: GGACAACTRCAAGCATCTAC	94	[17]
*Enterohemorrhagic Escherichia coli (EHEC)*	Shiga toxin 1 (*stx1*)	F: GCAAAGAMGTATGTWGATTCG R: GWGCCACTATCAATCATCAG	107	[17]
*Enterohemorrhagic Escherichia coli (EHEC)*	Shiga toxin 2 (*stx2*)	F: AATGCAAATCAGTCGTCAC R: TGCATCTCTGGTCATTGTAT	82	[17]
*Klebsiella pneumoniae*	Nitrogen regulation (*ntrA*)	F: CATCTCGATCTGCTGGCCAA R: GCGCGGATCCAGCGATTGGA	90	[18]
*Salmonella enterica*	Invasion gene (*invA*)	F: TCGTCATTCCATTACCTACC R: AAACGTTGAAAAACTGAGGA	119	[19]
*Shigella spp*.*/* *Enteroinvasive Escherichia coli (EIEC)*	Invasion plasmid antigen H (*ipaH*)	F: ACCGGCGCTCTGCTCTC R: GCAATGTCCTCCAGAATTTCG	62	[20]

### Real-time PCR

DNA extracted from filters was diluted tenfold and hundredfold in milliQ water to control for putative inhibitors. Real time PCR reactions were run in duplicates for all dilutions on 96-well plates (Applied Biosystems) with a total volume of 20 μL in each reaction. The PCR mix contained 10 μL SYBR Green Real-time PCR Master Mix (Applied Biosystems), 10 pmole of each primer, 6 μL water (DNAse- and RNAse-free) and 2 μL of sample. Two negative controls with duplicates were included in each plate. Negative controls consisted of milliQ water blanks since the high level of contamination in this watershed made it impossible to use filtrated water as a negative control for qPCR runs. Positive controls consisting of DNA from reference strains and a standard curve were included in duplicates in each PCR run.

Standard curves were made from purified PCR products using primers for each pathogen tested. The concentration for each purified PCR product was determined by measuring the OD_260_ in a NanoDrop spectrophotometer (NanoDrop Technologies, Wilmington, DE, USA), and the molar concentration was calculated using the molecular mass of the product and Avogadro’s number as described previously [21]. The standard curve was made by tenfold serial dilution in milliQ water from 10^6^ down to 10^1^ copies/μL. PCR standard curves were used to quantify the amount of gene copies in the qPCR reaction as described previously [22]. The copy number of each pathogen per 100 mL of collected river water was calculated by the assumption that one gene copy equals to one bacterium and by multiplying for dilution factors in the DNA extraction procedure, taking into account that 1/4 of the filter was used for extraction and the specific initial volume of river water that was filtrated through the filter.

Real-time PCR was performed on a 7500 real-time PCR instrument from Applied Biosystems, using the instrument’s default PCR program, but with 45 cycles of amplification, followed by melting curve analysis. Determination of threshold cycle (C_T_), number of gene copies and melting temperature (T_m_) was performed using the instrument software and default settings. The copy number per reaction was calculated using the settings for absolute quantification and the standard curve. All dilutions and standard curves were visually inspected and confirmed to show accurate dilutions (i.e. the C_T_ values should increase by approximately 3,3 cycles per tenfold dilution). The T_m_ value was recorded for each qPCR product, only samples with one peak corresponding to the expected T_m_ value were considered and T_m_ values were only allowed to vary 0.5°C above or below the standard curve T_m_ to be regarded as true positive readings.

### PCR detection of selected ESBL and carbapenem resistance associated genes

Gram-negative viable bacteria from fresh water, vegetables and soil samples were isolated in Mac Conkey agar during a previous study [10]. Five lactose fermenting and five non-lactose fermenting colonies per plate were tested for antibiotic resistance to 11 antibiotics by the disk diffusion method. The antibiotics used were: ampicillin (AM 10 μg), ampicillin-sulbactam (SAM 10 μg/10 μg), cefoxitin (FOX 30 μg), cefotaxime (CTX 30 μg), ciprofloxacin (5 μg), chloramphenicol (C 30 μg), gentamicin (CN 10 μg), nalidixic acid (NAL 30 μg), streptomycin (S 10 μg), tetracycline (TE 30 μg) and trimethoprim–sulphamethoxazole (SXT 1.25 μg /23.75 μg). Multi-resistance was defined as the resistance to three or more antibiotics. This collection of isolates is still under analysis and complete data will be included in future publications. In the present study, a sub-set of multi-drug resistant isolates out of the La Paz River collection was analysed by PCR for presence of selected ESBL and carbapenem resistance genes.

Multi-resistant bacterial isolates were grown overnight on LB media. Two to three colonies per isolate were re-suspended separately in tri-distilled water and subjected to boiling for DNA extraction. The primers for detection of ESBL and carbapenemase genes were designed in this study using the primer3 software (http://primer3.ut.ee), including the following gene families *bla*_CTX-M_ F: 5’-TGATACCACTTCACCTCGGG-3’, R: 5’-GCTTTACCCAGCGTCAGATT-3’; *bla*_KPC_ F: 5’-ACTGTAAGTTACCGCGCTGA-3’, R: 5’-AAGAAAGCCCTTGAATGAGCT; *bla*_NDM_ F: 5’-TGGCCCGCTCAAGGTATTTT-3’, R: 5’-GCCTTGCTGTCCTTGATCAG-3’; *bla*_VIM_ F: 5’-CTCGCGGAGATTGAAAAGCA-3’, R: 5’-CGRTCGTCATGRAAGTGCG-3’ and *bla*_OXA-48_ F: 5’-TATCGGCTGTGTTTTTGGTG-3’, R: 5’-CAACTTTTGTTTTCTTGCCATTC-3’. Positive and negative control strains were included in the PCR analysis and the PCR products were visualised in agarose gels under UV light to confirm the quality and size of the products.

### Whole genome sequencing (WGS) and annotation

The four isolates identified as positive for ESBL encoding genes were selected for whole genome sequencing and the identification of bacterial species, plasmid carriage and ARGs in the genomes, as well as multilocus sequence typing (MLST). One small loop of -80°C frozen stock vials were plated on LB-agar plates and incubated for 24 h at 37°C. One large bacterial colony from each plate was selected and washed in 300 μL milliQ water, where after the bacterial DNA was extracted using the DNeasy Blood & Tissue Kit from Qiagen according to the manufacturer’s instructions. The DNA concentration was measured using a Qubit 2.0 Fluorometer (Invitrogen). Sequencing libraries were prepared using the TruSeq Nano kit (Illumina, San Diego, CA) with a mean fragment length of 900 bp. Libraries were sequenced on the MiSeq platform v.3 chemistry, 2*300 bp, generating a coverage of >100X for all strains.

Illumina raw reads were trimmed and filtered using TrimGalore! Software v 0.3.7 (http://www.bioinformatics.babraham.ac.uk/projects/trim_galore/) applying the quality cut-off Q30, and only keeping reads longer than 30 bp. Filtered reads were *de novo* assembled using SPAdes v 3.10.1 [23]. The resulting draft and complete genomes were annotated with the *prokka* annotation pipeline v 1.1.12b [24] using the E24377A (CP000800.1) ETEC proteome as primary annotation source. Summary statistics from the sequencing, assembly, and annotation was collected using MultiQC v1.0 [25]. To perform initial functional analysis we used the CGE pipeline v 1.1 [26] which performs gene prediction using ResFinder [27], VirulenceFinder [28], *in silico* MLST typing, plasmid prediction and pMLST. Resistance gene prediction was also performed using the CARD [29] and Resqu [30] databases.

### Statistical analysis

Statistical analysis was performed using IBM SPSS Statistics Software version 22 considering P-values ≤0.05 as significant. One-Way ANOVA with Tukey as Post-Hoc Test was used to evaluate the statistical differences in number of pathogen genes between sampling points. Differences between sampling times were also evaluated (dry vs. rainy season, comparison of trimesters along the sampling year, etc.). Stepwise Linear Regression Analysis was used to assess the associations between physical-chemical parameters and DNA concentration, pathogen gene levels. The Stepwise model selects just the variables that are significantly associated with the dependent variable. The variables included in the model significantly explain a proportion of the variance observed in the dependent variable. The other, not associated, variables are excluded from the Linear Regression Model. For soil and vegetable samples, the difference on gene levels between dry and rainy season were evaluated using Mann-Whitney Test p≤0.05 in Graph Pad Prism version 6.

## Results

### Urban and hospital discharge increase the levels of enterobacteria, pathogenic *E*. *coli* and *Shigella spp*. in river water

Water samples collected monthly from sampling points (SPs) 1–4 in the La Paz River basin from April 2013 to March 2014 [10] were filtrated and total DNA extracted from the filters.

**Fig 1:**
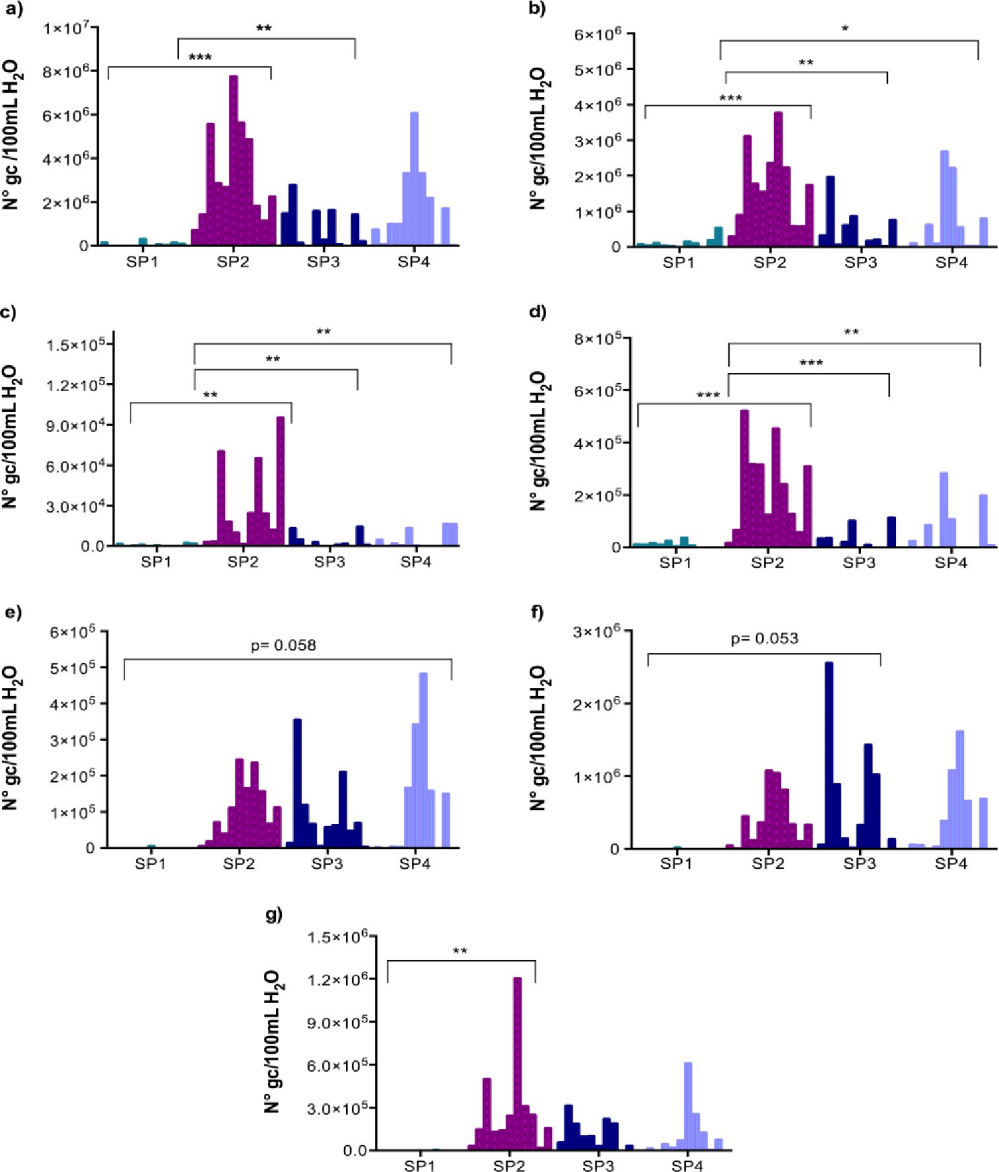
Quantification results for diarrheal bacterial pathogens in water samples from the La Paz River basin. a) enterobacteria-*gapA* b) ETEC-*eltB* c) ETEC- *estA1* d) ETEC- *estA2-4* e) EPEC/EHEC-*eae* f) EAEC-*aggR* g) *Shigella spp*./EIEC-*ipaH*. The bars show the number of copies per pathogen gene per 100 ml of river water (N° gc/100mL H_2_O) per month (April to March 2013–14, January results are absent) and per site obtained by qPCR absolute quantification analysis. All sampling points along the La Paz River basin are listed and compared. SP1: un-impacted site close to a water reservoir, SP2: site located in the Choqueyapu River and in the urban area, directly downstream of hospitals, SP3: agricultural area where river water is used for irrigation of crops and SP4: tributary river inside the urban area of La Paz city. Statistical significant differences between sampling points are indicated with stars, p≤0.05 (*), p≤0.01 (**) and p≤0.001 (***). Statistical significance analyzed by One-Way ANOVA, Post-Hoc Test: Tukey in SPSS.

Quantitative real-time qPCR assays were set up and evaluated for presence of the housekeeping gene *gapA* as an indicator of enterobacterial contamination. The samples were also investigated for the presence of different categories of diarrheagenic *E*. *coli* (enterotoxigenic *E*. *coli* (ETEC), enteropathogenic *E*. *coli* (EPEC), enteroaggregative *E*. *coli* (EAEC), enterohaemorhagic *E*. *coli* (EHEC) and enteroinvasive *E*. *coli* (EIEC)), *K*. *pneumoniae*, *S*. *enterica and Shigella spp*. by targeting signature genes (Table 1). The water samples were found to contain DNA from all the analyzed pathogens with the exception of shiga-like toxin 2 (*stx2*) positive EHEC. Bacteria positive for *gapA* were detected in 100% of river water samples and was the most abundant gene detected, followed by the gene encoding the ETEC heat labile toxin B chain (*eltB*) (Fig 1).

The first sampling point (SP1), recognized as a pristine site, was significantly different from the other three sites and contained significantly lower levels of all pathogen genes (Fig 1). Compared to SP1, gene levels of *gapA*, *eltB* and ETEC heat-stable toxins *estA1 and estA2-4* were significantly higher in the second sampling point located at an urban site (SP2) (p<0.05), with levels greater than 7x10^6^, 3.5x10^6^, 9x10^4^ and 5x10^5^ gene copies (gc) per 100 mL of river water, respectively. On the other hand, gene levels of EPEC/EHEC intimin *eae*, EAEC enteroaggregative regulator *aggR*, EHEC shiga-like toxin 1 *stx1*, *K*. *pneumoniae nitrogen regulator ntrA*, *S*. *enterica* invasion gene *invA* and *Shigella spp*.*/EIEC* invasion plasmid antigen H *ipaH* were not significantly enriched at SP2 compared to SP1 (p>0.05). These results suggest that urban and hospital discharge in La Paz contains high levels of enterobacteria and ETEC that might contaminate the Choqueyapu River.

High gene levels of *gapA* were also detected downstream in river water from the agricultural area (SP3) (up to 3x10^6^ gc/100 mL) and in the tributary river Jillusaya (SP4) (up to 6x10^6^ gc/100 mL) (Fig 1). SP4 presented the highest amount of *eae* copies during the year with a peak of approximately 5x10^5^ gc/100 mL in October 2013. However, the increase of *eae* gene copies from SP1 to SP4 was not statistically significant (p = 0.058). The agricultural area (SP3) generally had lower maximum levels of pathogen gene copies per month compared to the two urban sampling sites SP2 and SP4 with the exception of the *aggR gene* (Fig 1). High *aggR* levels were found in river water used for irrigation of fresh produce with a peak of approximately 2.5x10^6^ gc/100 mL in May 2013 (Fig 1). The presence of *ntrA* and *invA* genes was ubiquitous in all samples; the levels of these two genes did not show any significant difference between sampling points over the year (S1 Fig). We only detected positive samples for the *stx1* gene in SP2, SP3 and SP4 without significant differences between sampling sites (S1 Fig). Using One-Way ANOVA, no significant differences were found between months, trimesters and dry-rainy season during the year of sampling for any of the genes tested.

### Water conductivity is positively associated to DNA levels, amount of *gapA*-positive bacteria and pathogenic *E*. *coli* while water temperature is negatively associated

**Fig 2:**
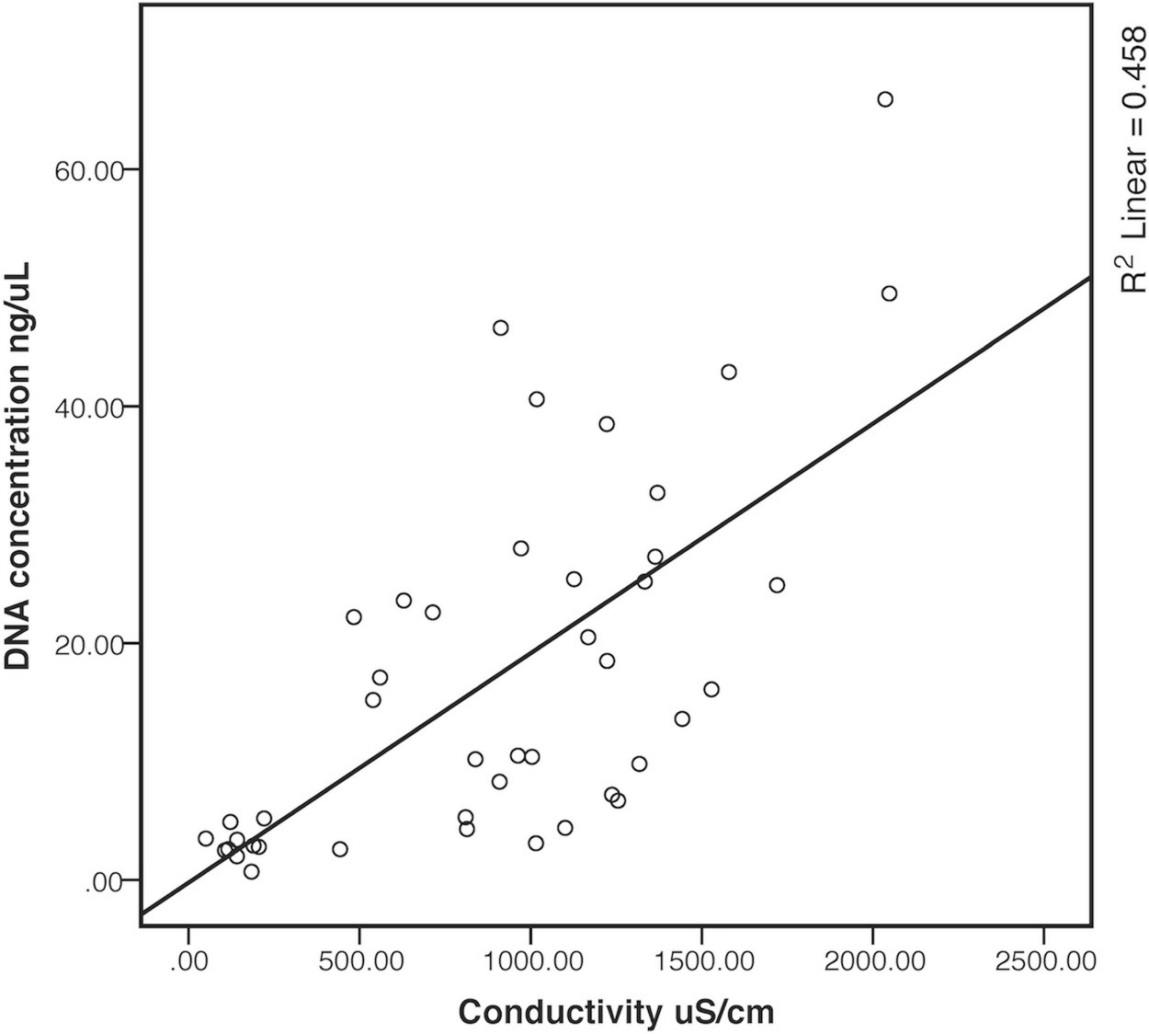
Linear association between conductivity (μS/cm) and DNA concentration (ng/μL) obtained from water samples from the La Paz River basin. Figure obtained from Linear Regression Analysis in SPSS version 22, p<0.01.

To search for associations between DNA levels, pathogen abundances and physical/chemical parameters, a Stepwise Linear Regression Analysis was performed. Variables such as pH, conductivity, temperature, redox potential and precipitation were included as independent variables for the analysis. The DNA concentration varied significantly between collection sites. Low levels were found in SP1 while SP2 and SP4 had higher levels. Regression analysis showed that DNA concentration was positively and linearly associated to conductivity (R^2^ = 0.46 and p<0.01) (Fig 2). Furthermore, water conductivity was positively associated with increased gene levels of *gapA*, *eltB*, *sth*, *aggR*, *eae*, *stx1*, *ntrA* and *ipaH* (Table 2). When water temperature was associated with pathogen gene levels, a negative association was observed for *gapA*, *eltB and estA2-4*. Hence lower temperatures and higher conductivity values significantly increase levels of enterobacteria and ETEC in the Choqueyapu River (Table 2).

**Table 2:** Stepwise linear regression results for all pathogens analyzed in water samples from La Paz River basin.

Pathogen	Target Gene	R^a^	R^2^ ^b^	P value	Variables Included in the Model (+/-)^c^
*Enterobacteriaceae*	Glyceraldehyde 3-phosphate dehydrogenase-A (*gapA*)	0.583	0.340	<0.001	Conductivity (+) Temperature (-)
Enterotoxigenic *Escherichia coli (ETEC)*	Heat-labile toxin B chain (e*ltB*)	0.636	0.405	<0.001	Conductivity (+) Temperature (-)
Enterotoxigeni*c Escherichia coli (ETEC)*	Heat-stable toxin (*estA1*)	NSC	NSC	NSC	NSC
Enterotoxigeni*c Escherichia coli (ETEC)*	Heat-stable toxin (*estA2-4)*	0.560	0.313	0.001	Conductivity (+) Temperature (-)
Enteropathogenic *Escherichia coli (EPEC)/* Enterohemorrhagic *Escherichia coli (EHEC)*	Intimin (*eae*)	0.451	0.203	0.002	Conductivity (+)
Enteroaggregative *Escherichia coli (EAEC)*	Enteroaggregative regulator (*aggR*)	0.540	0.292	<0.001	Conductivity (+)
Enterohemorrhagic *Escherichia coli (EHEC)*	Shiga toxin 1 (*stx1*)	0.393	0.155	0.009	Conductivity (+)
*Klebsiella pneumonia*	Nitrogen regulation (*ntrA*)	0.378	0.143	0.012	Conductivity (+)
*Salmonella enterica*	Invasion gene (*invA*)	NSC	NSC	NSC	NSC
*Shigella spp*.*/* Enteroinvasive *Escherichia coli (EIEC)*	Invasion plasmid antigen H (*ipaH*)	0.433	0.188	0.004	Conductivity (+)

^a^R: Regression Coefficient

^b^R^2^: Coefficient of Determination, percentage of the variance in the dependent variable explained by the independent variables collectively.

^c^(+/-): Direction of the linear association found using Stepwise Linear Regression Analysis with SPSS.

### Agricultural soils and farmed crops harbor the same pathogenic bacteria as contaminated irrigation water

**Fig 3:**
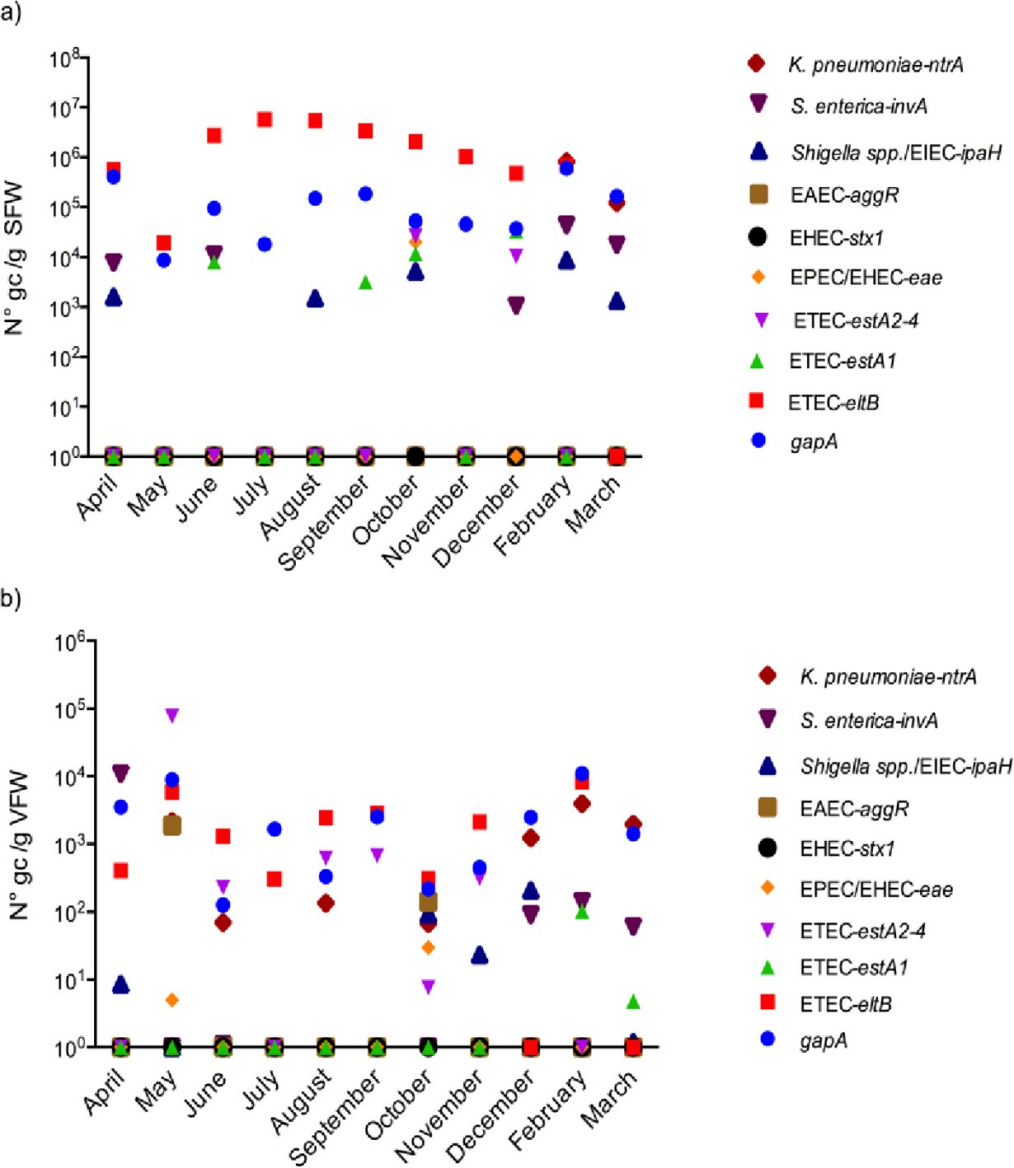
Number of pathogen gene copies in samples from the agricultural area Mecapaca (SP3) during the year of sampling (2013–14) obtained by qPCR absolute quantification analysis. a) soil samples and b) vegetables (lettuce and chard). Results are expressed in number of genes copies per gram of soil fresh weight (N° gc/g SFW) or vegetable fresh weight (N° gc/g VFW). Number of gene copies is expressed in logarithmic scale; only values greater than zero are plotted. Samples from January 2014 were not included in this study.

Soil and vegetable (lettuce and chard) samples were collected from the agricultural downstream area Mecapaca (SP3). Real-time qPCR analysis of DNA from soil samples showed the presence of eight out of 11 pathogen genes tested, where *gapA* and *eltB* were the most abundant and frequently detected genes (Fig 3). For *gapA* and *eltB the* numbers ranged between1x10^4^ to 1x10^6^ and 10 to 1x10^7^ gene copies per gram of soil fresh weight (gc/g SFW) respectively. Levels of *eltB* gene were higher than any other gene analyzed in soil samples with the highest peak registered during July-August 2013 (Fig 3). Pathogen genes such as *ntrA*, *invA*, *estA2-4*, *estA1*, *ipaH* and *eae* in decreasing order were detected in the samples. No significant difference was observed between dry (April-September) and rainy (October-March) season for all pathogen genes evaluated in soil samples (Mann-Whitney Test p≤0.05). Finally, *aggR*, *stx1* and *stx2* genes could not be found in soil samples.

Real-time qPCR analysis of DNA from filtrated vegetables rinse water revealed the presence of nine out of 11 pathogen genes tested. In this case, *gapA* and *eltB* were also the most abundant and frequently detected genes over the year, with numbers ranging from 1x10^2^ to 1x10^4^ and 1 to 1x10^4^ gene copies per gram of vegetable fresh weight (gc/g VFW), respectively. The highest peaks for both pathogen genes were found in May 2013 and February 2014 (Fig 3). Other pathogen genes such as *estA2-4*, *invA*, *ntrA*, *aggR*, *ipaH*, *eae* and *estA1* in decreasing order were also present in vegetable samples. No significant difference was observed between dry and rainy season for all pathogen genes evaluated with exception of *ipaH* gene, which was significant increased during the rainy season (p = 0.04). In vegetables, estA2-4 and *ntrA* genes were more often detected but in lower numbers than in soil samples (Fig 3). The *estA1 gene* was found in very low numbers during the year and *stx1*, *stx2* genes could not be found in any vegetable samples analyzed. Overall, pathogen gene levels in vegetables were much lower than in soil samples.

### *E*. *coli* and *Enterobacter cloacae* isolates carrying selected extended-spectrum β-lactamases (ESBLs) genes are present in the Choqueyapu River

A set of 101 bacterial isolates obtained from the Choqueyapu River, soil and vegetables and previously determined as resistant to three or more antibiotics by the disc diffusion test (see Materials and Methods section) was screened by standard PCR using a panel of primers designed to detect selected ESBL and carbapenem resistance genes.

Five of 101 isolates were found to be positive for ESBL genes *bla*_CTX-M_ while no carbapenem resistance genes were detected by PCR. Four of these isolates were whole genome sequenced using Illumina MiSeq to identify species, plasmids and antibiotic resistance genes (ARGs) as well as the sequence type (STs) in the case of *E*. *coli*, using the CGE pipeline v 1.1. Sequencing data are shown in Table 3 and S1 Table.

WGS data from this study are available at genbank under the BioProject number PRJNA449816 and BioSample accessions numbers SAMN08918384, SAMN08918385, SAMN08918386, SAMN08918387.

**Table 3:** Whole genome sequencing (WGS) data obtained from the four multi-resistant isolates scored positive for *bla*_CTX-M_ in PCR and obtained from the La Paz River basin.

Isolate	Identification	Plasmid Carriage	Antibiotic Resistance Genes (ARGs)	MLST^a^
HN77 (SP2-W)^b^	*Enterobacter cloacae*	IncHI2A, IncHI2, TrfA	*Sul2*, *APH(3'')-Ib*, *APH(6)-Id*, *bla*_TEM-1_, *bla*_CTX-M-3_, *QnrB1*, *dfrA14*, *AAC(3)-Iic*, *bla*_OXA-1_, *AAC(6')-Ib-cr*, *aadA*, *Tet(A)*	——
HN80 (SP2-W)^b^	*Escherichia coli*	IncFII(pRSB107), IncFIA, IncFIB(AP001918), Col8282, ColpVC, Col156, Col(BS512)	*APH(3'')-Ib*, *APH(6)-Id*, *bla*_TEM-1_, *tetA*, *mphA*, *sul1*, *aadA5*, *dfrA17*, *ErmB*	ST648
SO61 (SP3-S)^c^	*Escherichia coli*	IncI1	*QnrS1*, *bla*_CTX-M-3_	ST162
SO63 (SP3-S)^c^	*Escherichia coli*	IncFIA, IncFIB(AP001918), IncFII, Col(MG828), Col(BS512)	*emrE*, *AAC(6')-Ib-cr*, *bla*_OXA-1_	ST410

^a^MLST: *E*. *coli* Multi Locus Sequence Type

^b^SP2-W: Second sampling point (Holguin)-Water sample

^c^SP3-S: Third sampling point (Mecapaca)-Soil sample

Two of the isolates: HN77 and HN80 were isolated from water in SP2 and the other two isolates SO61 and SO63 were isolated from agricultural soil samples in SP3. Three of the isolates were found to be *E*. *coli* (HN80, SO61 and SO63) and one *Enterobacter cloacae* (HN77). All isolates carried plasmids and *E*. *coli* isolates HN80 and SO63 carried a higher number and diversity of plasmids than the others (Table 3). Several ARGs were identified in these isolates including resistance genes for tetracycline (*tet*(A)), sulfamethoxazole (*sul1* and *sul2*), quinolones (*qnrS1* and *qnrB1*), as well as beta-lactams including cephalosporins (*bla*_TEM,_ *bla*_*OXA*-1_ and *bla*_CTX-M-3_). *E*. *coli* isolates were identified by the CGE pipeline to belong to MLST types ST648, ST410 and ST162 (Table 3).

## Discussion

Water samples from different sites along the La Paz River basin were analyzed in order to detect and quantify the amount of diarrheal bacterial pathogens, in relation to physical and chemical parameters and seasonal variation. A positive linear association was found between conductivity and DNA concentration. A similar association was observed between conductivity and increasing numbers of enterobacteria, pathogenic *E*. *coli (*ETEC, EPEC, EHEC, EAEC, EIEC*)*, *K*. *pneumoniae* and *Shigella spp*. At the same time, a negative linear association between water temperature and increasing numbers of enterobacteria and ETEC was observed. Electrical conductivity (EC) measures the quantity of ionic salts dissolved in water, so changes in chemical composition of water bodies determine the variation in conductivity [31, 32]. On the other hand, EC can be used as a total dissolved solids (TDS) indicator [33]. The finding of a positive linear relation between EC and DNA concentration and number of pathogenic bacteria might indicate that dissolved particles, including bacteria, contribute to higher conductivity in water. Many approaches use EC as an indicator of chemical quality in natural and drinking water, where higher conductivity values are generally associated with higher amount of metals and other pollutants [31, 34–36]. Other studies have used EC as an indicator of bacterial presence in water. Galfi et al. [37] observed in storm-water and snowmelt from urban catchments in a Swedish city that temperature and pH were positively associated with the presence of total coliforms, *E*. *coli* and *Enterococci* but conductivity was shown to be negatively associated, presumptively due to salinity stress on bacterial survival. On the other hand, Lyew and Sheppard [38] reported an increase in EC in fed-batch columns due to the raise of metabolic activity in bacteria and the generation of charged metabolites. Overall, EC can be used as an indicator of contamination and bacterial presence in watersheds. However, the specific association is influenced by many other factors such as the geologic composition in the area, presence of substrates, bacterial species and other physical-chemical characteristics. In a preceding study, using the same samples Poma et al. [10] found that microbiological, physical and chemical parameters differ between impacted and un-impacted sites of sampling in the La Paz River basin. Using Principal Component Analysis (PCA), SP2, SP3 and SP4 clustered together and far from SP1, where the first two components including thermotolerant coliforms, conductivity, redox potential and pH accounted for approximately 98% of the observed variation.

Even though EC of drinking water is normally in the range of 50–500 μS/cm, it is hard to establish guidelines regarding conductivity and water usage because the measurement of this parameter also includes dissolved non-toxic salts [31]. For instance, it has been reported that humans can consume water with conductivity values in the range of 0–2500 μS/cm and it is also possible to use this water for irrigation of crops [34]. Different sampling sites along the La Paz River basin registered conductivity values from 50 μS/cm in the un-impacted area of Incachaca (SP1) up to 2050 μS/cm in the most contaminated point of the Choqueyapu River (SP2). These observations might suggest that conductivity can be used as an indicator of contamination and bacterial presence in water but that established guidelines for drinking and irrigation water have serious interpretation problems. The EC of a watershed not only mirrors its geologic conformation but also the presence of anthropogenic contaminants and the distinction between both sources is problematic and crucial for water monitoring programs [36].

In this study, different pathogens were evaluated in water samples from four sampling points along the La Paz River basin. Genes encoding enterobacteria and ETEC were the most abundant along the basin, they were significantly enriched in SP2, the most contaminated area when compared with the other sampling points. Pathogens such as EPEC, EHEC, EAEC, EIEC, *K*. *pneumoniae*, *S*. *enterica* and *Shigella spp*. were also detected along the sampling sites in this study. Ohno et al. [9] isolated pathogens such as ETEC, EPEC, EIEC and Salmonella O4 from water samples in the La Paz River and some other tributaries in the first report of diarrhea-causing bacteria in this basin. They also found that *E*. *coli* isolates from the river exhibited a markedly different survival rate compared with *E*. *coli* laboratory strains, being able to survive in river water longer periods of time. These observations support our findings and might suggest that the dominance of *E*. *coli* in the samples, specifically ETEC, might be due to a higher ability of survival in water. Poma et al. [10] preceded this study with a culture-dependent approach, which identified ETEC and *S*. *enterica* as the most frequently found enteropathogens in water samples from SP2, SP3 and SP4. No pathogens were detected in samples from SP1. In the present study, we were able to detect the genes for all pathogens tested, with exception of *stx2*-positive EHEC, in water from all sampling points including SP1. Even if the number of pathogen gene copies at SP1 was much lower in comparison with other sampling points. This difference could be attributed to the higher sensitivity of the molecular technique used in this study. Real-Time qPCR detects gene copies even at very low concentrations. Coinciding with the results reported by Poma et al. [10], our quantitative approach showed ETEC as the most abundant enteropathogen detected in all sampling points over the year. However, unlike the preceding study *S*. *enterica* was detected in lower numbers along the year and without a significant difference between sampling sites. Thus, the occurrence and abundance of *S*. *enterica* in the Choqueyapu River and its basin needs to be further investigated by other qualitative-quantitative methods.

Using One-Way ANOVA, no significant difference in number of pathogen genes was found between months, trimesters or dry-rainy season during the year of sampling (2013–14). This suggests a constant discharge of contaminants and pathogenic bacteria to the La Paz River basin across the year. Poma et al. [10] reported a significant difference in the numbers of thermotolerant coliforms between dry and rainy season (2.4x10^6^ vs. 4.4x10^5^ Most Probable Number (MPN) per 100 ml of water) in SP2, while the amount of thermotolerant coliforms in the other sampling points was similar between dry and rainy season. The difference could be attributed to the techniques applied for the identification and quantification of bacteria in environmental samples. In the present study, we used Real-Time qPCR, a technique with high specificity and sensitivity. However, one disadvantage of the technique is that it cannot differentiate between alive or dead bacteria [39] so it measures the total amount of bacteria in the watershed from both old and new events of contamination. Thus, the present study might provide a partially accumulative measure of bacteria in the Choqueyapu River and the La Paz River basin.

Analysis of diarrheal bacterial pathogens was also performed in agricultural soils and vegetables rinse water from SP3. Both compartments showed enterobacteria and ETEC as the most abundant, concurrent with results from water samples. Nevertheless, the number of gene copies was much higher in soils (gc/g SFW) than in vegetables (gc/g VFW), suggesting a possible accumulation process characteristic of soil ecosystems. All pathogens evaluated, with the exception of *stx1* and *stx2* positive EHEC and EAEC were detected in agricultural soils. In vegetables all pathogens evaluated were detected with exception of *stx1* and *stx2* positive EHEC. Therefore, a risk of transmission of diarrheal diseases to the population by the consumption of fresh produce must be considered. Ohno et al. [9] identified the presence of *Aeromonas caviae*, *Aeromonas spp*., *Chromobacterium violaceum* and *E*. *coli* as possible pathogenic strains in vegetables bought at local markets in La Paz, which were presumptively irrigated with contaminated water from the La Paz River basin. The preceding study by Poma et al. [10] measured the density of thermotolerant coliforms in the same set of soil and vegetable samples used in our study reporting 7.51x10^2^ and 1.46x10^2^ MPN/g respectively. In the case of soil the most frequent enteropathogen detected was ETEC (67%) followed by EAEC (33%), EIEC (33%), *Salmonella* (33%) and EPEC (25%). The most frequently found enteropathogen in vegetables was also ETEC (67%) followed by *Salmonella* (33%) and EAEC (17%). In our study, the most frequently found enteropathogen in vegetables was also ETEC carrying different type of toxin genes (*eltB*, *estA2-4* and estA1) in variable proportions. This might be attributable to 1) ETEC strains with different toxin profiles potentially have different ability to survive in water and be transmitted to fresh produce or 2) ETEC strains with different toxin profile attach to the surface of vegetables in different manners and in some cases do not rinse off. We have in fact previously shown that ETEC strains have different capacity to adhere to rocket salad leaves [7]. However, no obvious pattern of toxin expression could be inferred from that study and hence this needs to be further investigated. In vegetables not being disinfected the number of many diarrheal bacterial pathogens evaluated in this study would be close to the lowest infectious dose and hence pose a plausible risk of disease [40].

In the study performed by Gonzales et al. [41], ETEC was identified as the second most prevalent DEC in fecal samples from children with diarrhea in hospitals from main cities in Bolivia. ETEC (6.6%) was preceded by EAEC (11.2%) and followed by EPEC and EIEC-EHEC (5.8% and <1%). In our study, the three most prevalent DEC categories in Bolivian children with diarrhea were detected and quantified in water, soil and vegetable samples from the La Paz River basin. These findings might suggest that specific DEC categories are circulating among the population, they are discharged to the river and they might be transmitted again from the water body to the population in a cyclic way. Many other studies have shown that presence of pathogens in water coincide with disease [42, 43]. However, the inference of directionality in this infectious cycle necessarily involves further studies and evaluation.

The present study identified three *E*. *coli* and one *Enterobacter cloacae* isolates in water from SP2 and soil from SP3 in the La Paz River basin. Whole genome sequencing and subsequent analyses of the isolates confirmed the presence of different conjugative plasmids including IncF, IncI and IncH groups. These are among the most commonly reported “epidemic plasmids” isolated from humans, animals and the environment and may carry ESBL and carbapenemase genes [44]. Diverse antibiotic resistance genes were found in the four sequenced isolates including ESBL genes such as bla_OXA-1_, bla_TEM-1_ and *bla*_CTX-M-3_. However, no carbapenem resistance genes were found in our study. ESBLs are well recognized for their ability to inactivate extended-spectrum cephalosporins and monobactams. Genes encoding these hydrolyzing enzymes, especially the *bla*_CTX-M_ group, are distributed in bacteria almost everywhere. Thus, ESBLs are being considered a worldwide pandemic causing serious problems for infection control [45–47]. In South America, many studies have reported *bla*_CTX-M_ positive bacteria in human isolates [48–50] as well as in animal hosts [51–55]. In Bolivia, bacterial isolates carrying ESBLs, commonly *bla*_CTX-M-2_ and *bla*_CTX-M-15_ among others have been detected in clinical and fecal samples [56–58]. Moreover, clinical bacterial isolates with carbapenem resistance (CRE) have been reported in different countries of Latin America and the Caribbean [59] including Bolivia [60, 61].

Aquatic and agricultural compartments have also shown the presence of *bla*_CTX-M_ carrying bacteria in many parts of the world [62–69]. In South America, the presence of *bla*_CTX-M-1_, bla_CTX-M-2_, bla_CTX-M-8,_ bla_CTXM-9_ and bla_CTX-M-15_ among others, have been reported in wastewater, hospital sewage, lakes and farming soils in Brazil [70–73]. Interestingly, *bla*_CTX-M-15_ carrying bacteria were also found in vegetables from the market in Ecuador [74]. In Bolivia, the reports of ARGs in environmental samples are scarce, only high occurrence of sulfonamide resistance genes in the Katari watershed of the Titicaca Lake has been reported before [75]. To our knowledge, the present study is the first report of environmental bacterial isolates carrying ESBLs such as bla_OXA-1_, bla_TEM-1_ and *bla*_CTX-M-3_ in Bolivia. However, among a set of multi-resistant bacteria to commonly used antibiotics we detected a low frequency of the ESBL *bla*_CTX-M_ gene (4.95%). This might be explained by the fact that the set of bacterial isolates tested for ESBLs and CRE genes were first isolated by culture and tested by disc diffusion tests for resistance to at least 3 of 11 antibiotics, where the majority were not ESBL or carbapenem antibiotics. Therefore, the low frequency detected might be due to the fact that this study only evaluated the presence of selected ESBL and carbapenem resistance genes in a set of already defined multi-resistant isolates. The four *bla*_CTX-M_ positive isolates were re-tested by disc diffusion tests for resistance to Cefotaxime (CTX) and three were phenotypically resistant, indicating one silent gene. For this reason, future studies in the La Paz River basin should consider a broader analysis of ESBLs and CRE determinants using both genotypic and phenotypic methods.

The *E*. *coli* isolates sequenced in this study were found to belong to the previously described MLST types ST648, ST410, and ST162 that are repeatedly found in hospitals, animals and the environment. Interestingly, ST648 and ST410 are globally emerging *E*. *coli* MLST types known to sometimes carry *bla*_CTX-M_, *bla*_OXA_ and even *bla*_NDM_ genes [76–78]. ST648 and ST410 carrying *bla*_CTX-M_ genes have previously been found in free-roaming cats in Brazil [54] and fresh vegetables in a municipal market from Ecuador [74]. ST162 is also emerging as an ESBL and multi-resistant clone. [79]. The presence of these MLST types in water and soil could hence pose a risk of transmission to humans and animals via the environment. In this sense, a “One Health” concept has been proposed in order to tackle emergence and dissemination of infectious diseases and antimicrobial resistant organisms worldwide. The One Health concept supports the idea of three different and interrelated interfaces between humans, animals, and the environment where surveillance and action strategies are necessary to mitigate the emergence and spread of infectious/drug-resistant microorganisms [80]. Control of agricultural sources to prevent release of infectious and resistant bacteria and treatment of domestic, industrial and hospital wastewater are the most important strategies raised to prevent spread of ARB and infectious diseases to the environment and society [81, 82].

In conclusion, this study evidences the risk of transmission of diarrheal diseases directly or indirectly from the Choqueyapu River and its basin due to the presence of diarrheal pathogens in river water, vegetables and agricultural soils. Bacterial isolates carrying ESBL genes and conjugative resistant plasmids obtained from the basin indicate that the risk is not only associated with the transmission of infectious bacteria, but also with the possibility of transmission of antibiotic resistant bacteria and the resistance genes they carry from the environment to the community. Our findings strongly point out the need of surveillance, control and management of this watershed.

## Supporting information

S1 Fig Quantification results for diarrheal bacterial pathogens in water samples from the La Paz River basin. a) EHEC-*stx1*, b) *S*.*enterica*-*invA* and c) *K*. *pneumoniae*-*ntrA*. The bars show the number of copies per pathogen gene per 100 ml of river water (N° gc/100mL H_2_O) per month (April to March 2013–14, January results are absent) and per site obtained by qPCR absolute quantification analysis. Number of gene copies is expressed in logarithmic scale. All sampling points along the La Paz River basin are listed and compared. SP1: un-impacted site close to a water reservoir, SP2: site located in the Choqueyapu River and in the urban area, directly downstream of hospitals, SP3: agricultural area where river water is used for irrigation of crops and SP4: tributary river inside the urban area of La Paz city.
(DOCX)

S1 Table Sequencing data from the four multi-resistant bacterial isolates from the Choqueyapu River positive for *bla_CTX-M_* in PCR. (DOCX)
