# Supplementary material for: Diarrheal bacterial pathogens and multi-resistant enterobacteria in the Choqueyapu River in La Paz, Bolivia
Source: PLoS One. 2019 Jan 14;14(1):e0210735. doi: 10.1371/journal.pone.0210735 (PMC6331111; doi:10.1371/journal.pone.0210735)
Supplement: S1 Fig — a) EHEC-stx1, b) S.enterica-invA and c) K. pneumoniae-ntrA. The bars show the number of copies per pathogen gene per 100 ml of river water (N° gc/100mL H2O) per month (April to March 2013–14, January results are absent) and per site obtained by qPCR absolute quantification analysis. Number of gene copies is expressed in logarithmic scale. All sampling points along the La Paz River basin are listed and compared. SP1: un-impacted site close to a water reservoir, SP2: site located in the Choqueyapu River and in the urban area, directly downstream of hospitals, SP3: agricultural area where river water is used for irrigation of crops and SP4: tributary river inside the urban area of La Paz city. (DOCX) [file pone.0210735.s001.docx]

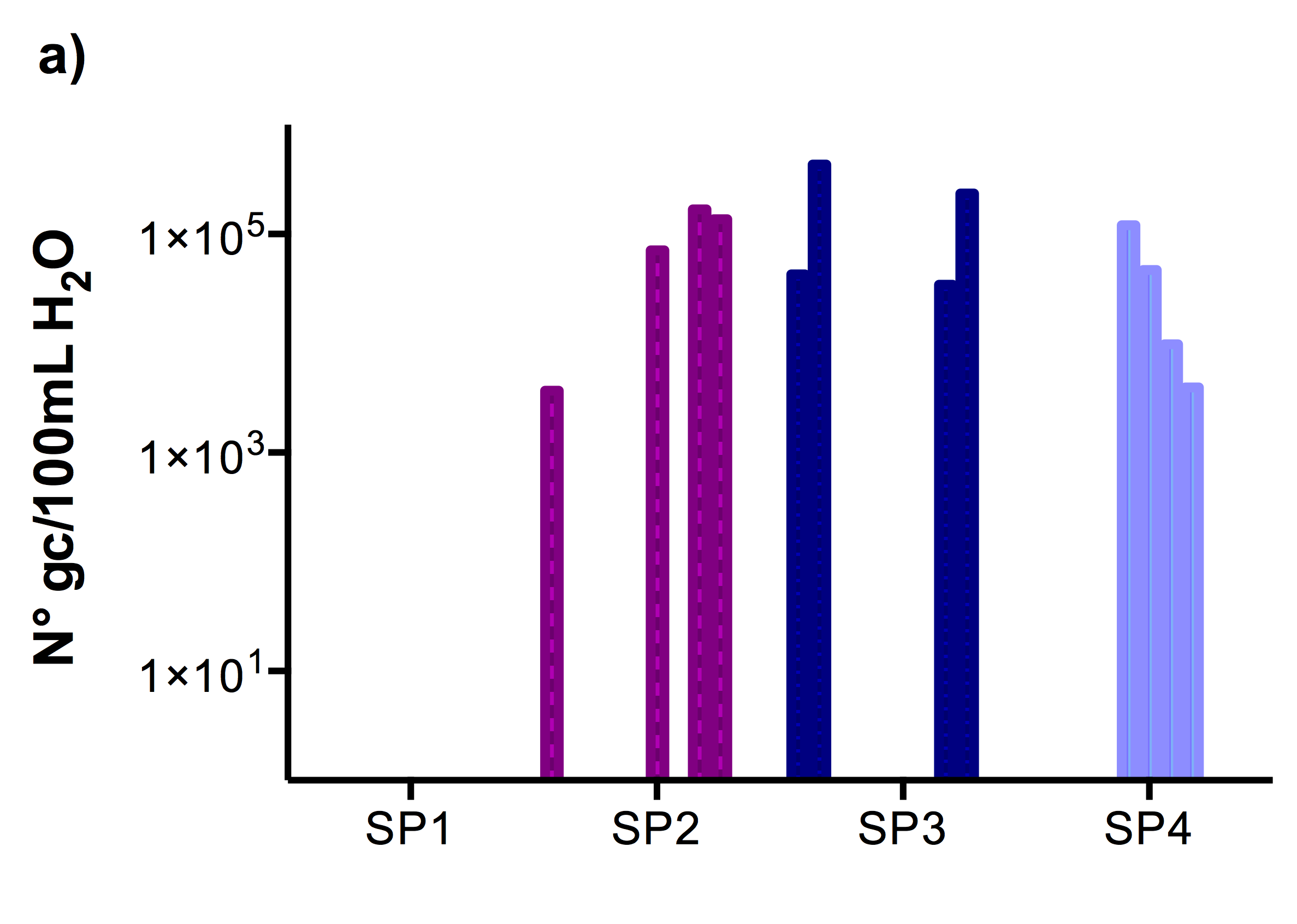

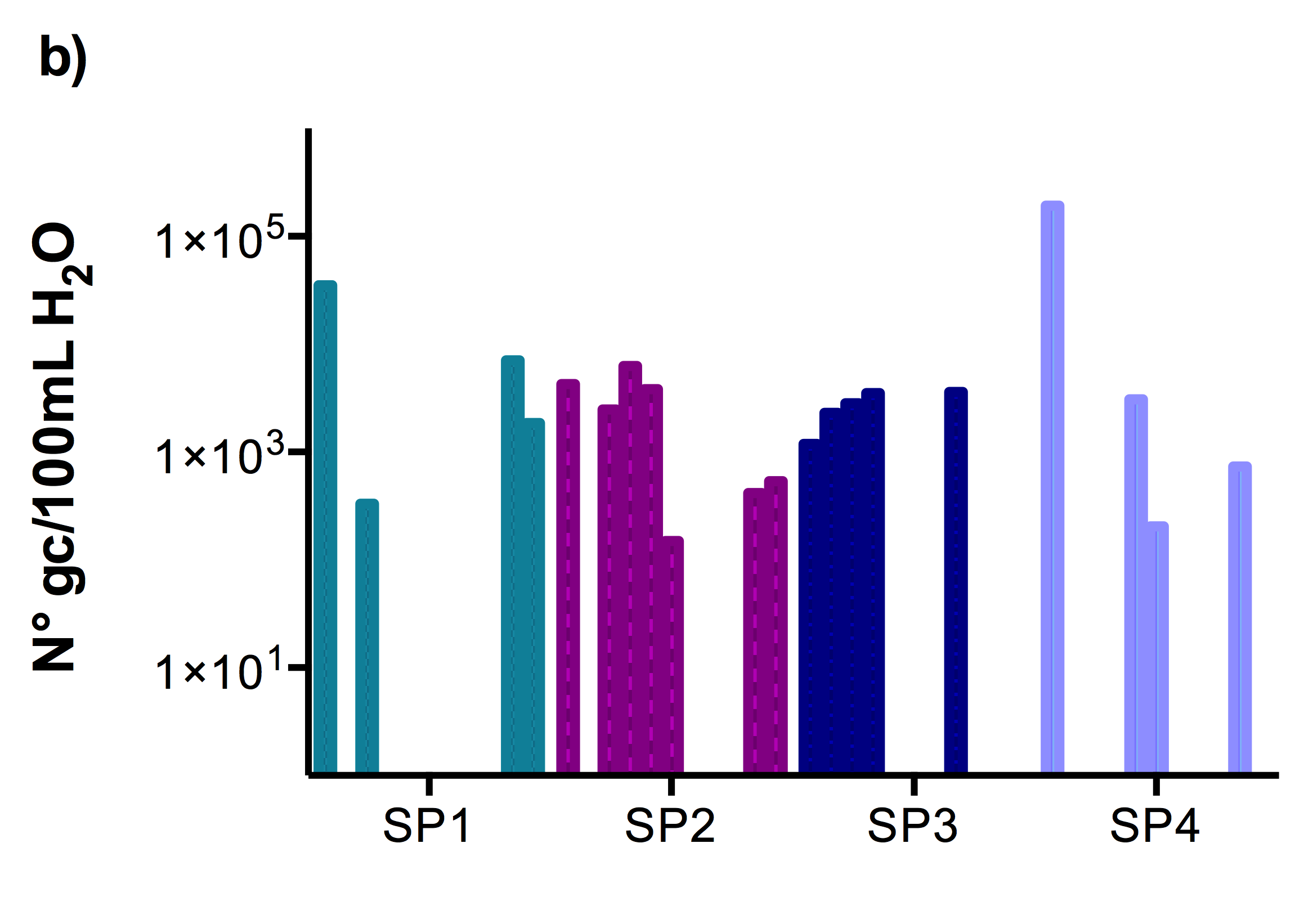


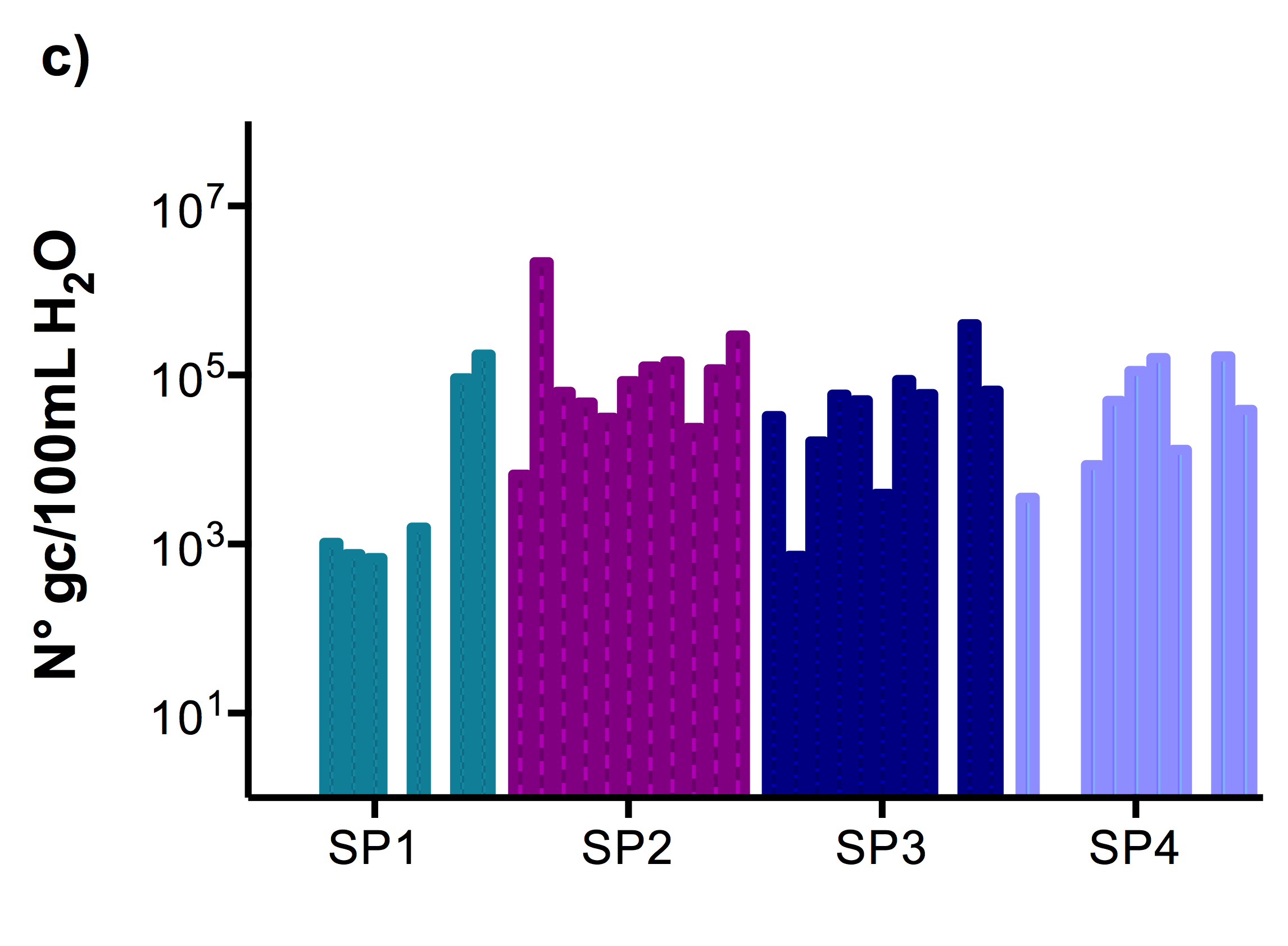


**S1 Fig. Quantification results for diarrheal bacterial pathogens in water samples from the La Paz River basin.** a) EHEC-*stx1,* b) *S.enterica*-*invA* and c) *K. pneumoniae*-*ntrA*. The bars show the number of copies per pathogen gene per 100 ml of river water (N° gc/100mL H_2_O) per month (April to March 2013-14, January results are absent) and per site obtained by qPCR absolute quantification analysis. Number of gene copies is expressed in logarithmic scale. All sampling points along the La Paz River basin are listed and compared. SP1: un-impacted site close to a water reservoir, SP2: site located in the Choqueyapu River and in the urban area, directly downstream of hospitals, SP3: agricultural area where river water is used for irrigation of crops and SP4: tributary river inside the urban area of La Paz city.
