# Supplementary material for: Diarrheal bacterial pathogens and multi-resistant enterobacteria in the Choqueyapu River in La Paz, Bolivia
Source: PLoS One. 2019 Jan 14;14(1):e0210735. doi: 10.1371/journal.pone.0210735 (PMC6331111; doi:10.1371/journal.pone.0210735)
Supplement: S1 Table — (DOCX) [file pone.0210735.s002.docx]

**Supporting Information**

**S1 Table. Sequencing data from the four multi-resistant bacterial isolates from the Choqueyapu River positive for *bla_CTX-M_* in PCR**

| **Isolate** | **Number of contigs** | **Total length** | **Min length** | **Max length** | **Mean length** | **Median length** | **N50** |
| --- | --- | --- | --- | --- | --- | --- | --- |
| HN77 | 37 | 4993863 | 728 | 2512134 | 134969,27 | 5026 | 2512134 |
| HN80 | 100 | 5384239 | 522 | 720746 | 53842,39 | 5507 | 268749 |
| SO61 | 80 | 4929446 | 532 | 429874 | 61618,08 | 20847 | 193433 |
| SO63 | 86 | 4756476 | 561 | 457545 | 55307,86 | 9847 | 206077 |
